# The association between loneliness and frailty among community-dwelling older adults in five European countries: a longitudinal study

**DOI:** 10.1093/ageing/afae210

**Published:** 2024-10-10

**Authors:** Lizhen Ye, Esmee Bally, Sophie A Korenhof, Irene Fierloos, Tamara Alhambra Borrás, Gary Clough, Hein Raat, Amy van Grieken

**Affiliations:** Department of Public Health, Erasmus MC—University Medical Center Rotterdam, Rotterdam, The Netherlands; Department of Public Health, Erasmus MC—University Medical Center Rotterdam, Rotterdam, The Netherlands; Department of Public Health, Erasmus MC—University Medical Center Rotterdam, Rotterdam, The Netherlands; Department of Public Health, Erasmus MC—University Medical Center Rotterdam, Rotterdam, The Netherlands; Polibienestar Research Institute—Universitat de València, Valencia, Spain; Department of Public Health, The University of Manchester, Manchester, UK; Department of Public Health, Erasmus MC—University Medical Center Rotterdam, Rotterdam, The Netherlands; Department of Public Health, Erasmus MC—University Medical Center Rotterdam, Rotterdam, The Netherlands

**Keywords:** emotional loneliness, social loneliness, physical frailty, psychological frailty, social frailty, older people

## Abstract

**Background:**

Loneliness is described as the subjective experience of unfulfilled personal and social needs, with emotional and social domains. Frailty is a state of vulnerability to stressors, which is often characterised by impairment in the physical, psychological and/or social domain.

**Objective:**

This study aims to examine the bidirectional association between loneliness and frailty across the different domains.

**Methods:**

The study included 1735 older adults from the Urban Health Centres Europe project. Loneliness was assessed using the six-item De Jong Gierveld Loneliness Scale. Frailty was assessed by the Tilburg Frailty Indicator. Multivariate linear regression and cross-lagged panel models were used to explore the associations between the social and emotional loneliness dimensions and overall, physical, psychological and social frailty.

**Results:**

A bidirectional association existed between overall loneliness and overall frailty (loneliness to frailty: β = 0.09, 95% CI: 0.03, 0.15; frailty to loneliness: β = 0.05, 95% CI: 0.004, 0.10). Higher levels of overall loneliness at baseline were associated with higher levels of psychological frailty at follow-up (β = 0.05, 95% CI: 0.00, 0.10). The reverse association was not significant. A bidirectional association existed between overall loneliness and social frailty (loneliness to social frailty: β = 0.05, 95% CI: 0.01, 0.10; social frailty to loneliness: β = 0.05, 95% CI: 0.00, 0.09).

**Conclusion:**

This study confirms the importance of addressing loneliness among older adults. Interventions that increase social support, exercise engagement and promote healthy behaviours may be effective in reducing the risk of frailty among older adults and simultaneously preventing loneliness.

## Key Points

- Social loneliness at baseline was significantly associated with higher levels of overall, physical and social frailty at follow-up.
- Bidirectional associations were found between overall loneliness and overall frailty, as well as between overall loneliness and social frailty, but not for physical and psychological frailty.
- Loneliness, particularly social loneliness, is an independent risk factor for overall, physical, psychological and social frailty among older adults.

## Introduction

Loneliness is described as the subjective experience of unfulfilled personal and social needs; it has both emotional and social dimensions [1]. Emotional loneliness refers to the feeling of being disconnected from others and lacking meaningful relationships [2], while social loneliness refers to a subjective experience of the absence of social contact or engagement with others [3]. Loneliness is a pervasive issue among older adults, who are generally characterised as those older than 65 years and are more sensitive to loneliness than younger adults [4]. Numerous chronic conditions have been reported to have associations with loneliness, including heart disease, lung disease, cardiovascular disease, hypertension, stroke and so on [5]. Additionally, loneliness may be associated with psychological problems, such as depression, psychological stress and anxiety [5]. It is also associated with morbidity and mortality [6, 7].

Frailty is a state of vulnerability to stressors due to decreased physical, cognitive and functional reserves [8]. It is a dynamic and multidimensional concept depicting impairments in the physical, psychological and/or social domains [9, 10]. Physical frailty refers to the presence of at least three of the following indicators: weakness, self-reported exhaustion, slow gait speed, unintentional weight loss and insufficient physical activity [11]. Psychological frailty refers to impairments in various areas, such as mood, cognition and incontinence [12]. Social frailty refers to a state of being at risk of losing or having already experienced the loss of resources that are essential for fulfilling one or more basic social demands [13]. Frailty is a prevalent condition among older adults, affecting 25%–50% of those aged 85 and older [8, 14]. Frail older adults are more prone to negative outcomes such as falls [15], disability [16], institutionalisation [17] and premature death [18], leading to higher healthcare usage and financial strain on the health system [19].

Although loneliness and frailty are independent concepts, they are significantly associated [20]. Studies show that higher levels of loneliness predict increased frailty over time [20, 21]. Conversely, frailty can exacerbate loneliness by limiting older adults’ participation in social activities and their ability to maintain social connections [22]. Both loneliness and frailty share common risk factors such as older age, chronic illness and limited social support, creating a reinforcing cycle [23]. Chronic illnesses such as cardiovascular disease and diabetes contribute to physical limitations, increasing the risk of frailty and social isolation [24]. Additionally, inadequate social support impedes physical activity and healthcare access, further compromising health [25]. Biologically, chronic inflammation and neuroendocrine dysregulation linked to loneliness [26] contribute to frailty. Behaviourally, loneliness leads to decreased physical activity, poorer nutrition and disrupted sleep, all of which contribute to frailty [27]. Psychologically, loneliness is associated with a higher risk of depression and anxiety, accelerating physical frailty and potentially impairing cognitive function and decision-making [28]. However, the precise nature and strength of these relationships, particularly concerning emotional versus social loneliness and diverse frailty domains, remain inadequately explored, suggesting a need for further investigation to inform targeted interventions.

In this context, our study aims to enhance the understanding of the association between loneliness and frailty, focusing on their potential bidirectional relationship and specific domains. By evaluating physical, psychological and social frailty, we seek to identify patterns and trends. Ultimately, our research aims to inform targeted interventions to prevent or mitigate the adverse effects of loneliness and frailty in older adults, improving overall health and well-being.

## Methods

### Study settings and participants

This study used data from the Urban Health Centres Europe (UHCE) project, which aimed to prevent loneliness, frailty, falls and inappropriate medication use in older adults [29]. Conducted in the Netherlands, Croatia, Spain, the UK and Greece, the UHCE project provided assessments and integrated care pathways for participants. Initial assessments focused on frailty, fall risks, polypharmacy and loneliness, with results discussed among the participant, a care coordinator and a physician to create a shared care plan. Participants were referred to evidence-based care pathways specific to their city, including multifactorial fall prevention, polypharmacy actions, loneliness support and frailty interventions [30].

The UHCE project included 2325 older adults who completed self-report questionnaires at baseline and 1-year follow-up from May 2015 to June 2017 [30]. Ethical review procedures were followed in all countries, with written informed consent obtained from all participants. The study was registered as ISRCTN52788952. More details on the study design have been described elsewhere [29–31].

This study included 1844 older adults who completed both baseline and follow-up questionnaires [29]. Participants with missing data (*n =* 109) on loneliness (overall, emotional and social) and frailty (overall, physical, psychological and social) were excluded, resulting in a final sample of 1735 older adults for analysis.

### Loneliness and frailty

Loneliness was assessed using the six-item De Jong Gierveld Loneliness Scale, which includes subscales for emotional and social loneliness [3, 32]. Emotional loneliness was measured by three items assessing feelings of emptiness, not having people around and rejection. Social loneliness was assessed by three items evaluating the feeling of having people to rely on, trust completely and feel close to. Each item had answer options of ‘no’ (score = 0), ‘more or less’ (score = 1) and ‘yes’ (score = 1). Scores for emotional and social loneliness range from 0 to 3, and overall loneliness scores range from 0 to 6, with higher scores indicating higher levels of loneliness. Participants with scores <2 were classified as not lonely, while those with scores ≥2 were classified as lonely [32].

Frailty was assessed using the Tilburg Frailty Indicator (TFI), a validated instrument for older adults [33]. The TFI consists of 15 self-report questions covering three domains: physical (eight items, score range 0–8), psychological (four items, score range 0–4) and social (three items, score range 0–3) frailty [34, 35]. The overall frailty score is the sum of these domain scores, ranging from 0 to 15 [34]. Participants with an overall frailty score of at least 5 were categorised as frail. The cut-off points for physical, psychological and social frailty were 3, 2 and 2, respectively [36]. These cut-offs are validated, as detailed in Gobbens *et al*. [34].

### Covariates

Following previous literature [8, 37, 38], several indicators were assessed and incorporated as covariates: age (years), sex, country, education, alcohol risk, exercise frequency, number of chronic conditions and health-related quality of life (HRQoL). Additionally, whether participants were assigned to the intervention group (yes/no) was included as a covariate in all models.

Education level was classified according to the highest completed level based on the 2011 International Standard Classification of Education (ISCED) and grouped into ‘primary or lower’, ‘secondary or equivalent’ and ‘tertiary or higher’ [39]. Alcohol risk was assessed using three items from the Alcohol Use Disorders Identification Test—Consumption, with scores ranging from 0 to 12 [29]. Participants were categorised as having no alcohol risk (score < 4 for men; score < 3 for women) or alcohol risk (score ≥4 for men; score ≥3 for women) [40]. Exercise frequency concerned the number of times participants engaged in activities that require low or moderate energy (once a week or less; more than once a week).

The number of chronic conditions was measured based on current or previous chronic conditions, including heart attack, hypertension, diabetes, stroke, high blood cholesterol, asthma, arthritis, osteoporosis, chronic lung disease, cancer or malignant tumour, stomach or duodenal ulcer, Parkinson’s disease, cataract and hip or femoral fracture [41]. HRQoL was measured using the 12-Item Short Form Survey (SF-12), a reliable and valid standardised instrument [42]. The SF-12 provides a Physical Component Summary (PCS-12) and a Mental Component Summary (MCS-12), with higher scores indicating higher levels of HRQoL [43].

### Statistical analysis

Descriptive statistics were used to summarise the study population’s characteristics. Continuous variables were reported as means and standard deviations (SD), while categorical variables were presented as frequencies and percentages.

Covariate descriptives were stratified by loneliness dimensions. Loneliness was assessed at baseline (yes/no), emotional and social loneliness (yes/no) and 12-month change in loneliness (categories: not lonely to lonely, continued not lonely, continued lonely, lonely to not lonely). T-tests, one-way analysis of variance and chi-squared tests evaluated these characteristics.

Unidirectional longitudinal associations between loneliness dimensions (overall, emotional and social at baseline; 12-month change in overall loneliness) and frailty (overall and by domain) were analysed using multivariate linear regression. Separate models were created for overall frailty and each frailty domain. Models were run in two sets: the first (crude) without covariate adjustments and the second adjusted for baseline covariates and the relevant frailty domain.

To explore potential bidirectional associations between overall loneliness and frailty, cross-lagged panel modelling (CLPM) was employed [44]. Wald tests evaluated differences between lagged coefficients. CLPM parameters were estimated using maximum likelihood with robust standard errors to handle data non-normality and full information maximum likelihood to address missing covariate values. Interaction effects by sex and country on loneliness were assessed, applying Bonferroni correction (*P* = .05/2 = .025) for multiple comparisons [45].

Descriptive and unidirectional analyses were conducted in the IBM SPSS Statistics for Windows (version 25 Armonk, NY, USA: IBM Corp). CLPM analysis was performed with the lavaan package (version 4.1.2; R Development Core Team) within the R Studio (version 2021.09.2X64 ENG).

## Results

### Baseline characteristics of participants

Table 1 presents the baseline characteristics of the study population. Participants had a mean age of 79.6 years (SD = 5.5), with 61.2% being female. Lonely participants were significantly older (*P* < .001), more often female (*P* < .001), more often from Croatia (*P* < .001). They were also less likely to have completed tertiary or higher education (*P* < .05), less likely to exercise more than once a week (*P* < .001), more likely to have a higher number of chronic conditions (*P* < .001) and reported lower mental and physical HRQoL (*P* < .001).

**Table 1:** General characteristics of the study population (*n =* 1735)

Covariates	Total (*n =* 1735)	Baseline loneliness		12-month change in loneliness			
		Not lonely (*n =* 933, 53.8%)	Lonely (*n =* 802, 48%)	Not lonely to lonely (*n =* 228, 13.1%)	Continued not lonely (*n =* 705, 40.6%)	Continued lonely (*n =* 605, 34.9%)	Lonely to not lonely (*n =* 197, 11.4%)
Age	79.6 ± 5.5	79.12 ± 5.51^a^	80.16 ± 5.51^a^	79.54 ± 5.40^b^	78.98 ± 5.55^b^	80.24 ± 5.40^b^	79.92 ± 5.84^b^
Sex							
Male	674 (38.8%)	399 (42.8%)^c^	275 (34.3%)^c^	87 (38.2%)^c^	312 (44.3%)^c^	202 (33.4%)^c^	73 (37.1%)^c^
Female	1061 (61.2%)	534 (57.2%)^c^	527 (65.7%)^c^	141(61.8%)^c^	393 (55.7%)^c^	403 (66.6%)^c^	124 (62.9%)^c^
Country							
The Netherlands	263 (15.2%)	163 (17.5%)^c^	100 (12.5%)^c^	32 (14.0%)^c^	131 (18.6%)^c^	65 (10.7%)^c^	35 (17.8%)^c^
Greece	224 (12.9%)	109 (11.7%)^c^	115 (14.3%)^c^	42 (18.4%)^c^	67 (9.5%)^c^	92 (15.2%)^c^	23 (11.7%)^c^
Croatia	420 (24.2%)	110 (11.8%)^c^	310 (38.7%)^c^	59 (25.9%)^c^	51 (7.2%)^c^	265 (43.8%)^c^	45 (22.8%)^c^
Spain	391 (22.5%)	248 (26.6%)^c^	143 (17.8%)^c^	42 (18.4%)^c^	206 (29.2%)^c^	91 (15.0%)^c^	52 (26.4%)^c^
The UK	437 (25.2%)	303 (32.5%)^c^	134 (16.7%)^c^	53 (23.2%)^c^	250 (35.5%)^c^	92 (15.2%)^c^	42 (21.3%)^c^
Education							
Primary or less	427 (24.6%)	227 (24.7%)^b^	200 (25.2%)^d^	61 (26.9%)	166 (24.0%)	147 (24.5%)	53 (27.3%)
Secondary	1113 (64.1%)	583 (63.4%)^d^	530 (66.8%)^d^	146 (64.3%)	437 (63.2%)	401 (66.9%)	129 (66.5%)
Tertiary or higher	172 (9.9%)	109 (11.9%)^b^	63 (7.9%)^d^	20 (8.8%)	89 (12.9%)	51 (8.5%)	12 (6.2%)
Missing	23 (1.3%)						
Alcohol risk							
No	1195 (68.9%)	627 (70.5%)^d^	568 (75.1%)^d^	159 (76.1%)^d^	468 (68.8%)^d^	429 (75.7%)^d^	139 (73.5%)^d^
Yes	450 (25.9%)	262 (29.5%)^d^	188 (24.9%)^d^	50 (23.9%)^d^	212 (31.2%)^d^	138 (24.3%)^d^	50 (26.5%)^d^
Missing	90 (5.2%)						
Exercise							
More than once a week	1265 (72.9%)	749 (80.8%)^c^	516 (64.7%)^c^	163 (71.8%)^c^	586 (83.7%)^c^	376 (62.6%)^c^	140 (71.4%)^c^
Once a week or less	459 (26.5%)	178 (19.2%)^c^	281 (35.3%)^c^	64 (28.2%)^c^	114 (16.3%)^c^	225 (37.4%)^c^	56 (28.6%)^c^
Missing	11 (0.6%)						
Multimorbidity (score)	3.90 ± 1.88	3.70 ± 1.80^a^	4.12 ± 1.95^a^	3.82 ± 1.85^b^	3.66 ± 1.78^b^	4.23 ± 1.96^b^	3.80 ± 1.91^b^
Health-related quality of life							
Mental	42.05 ± 11.93	54.82 ± 8.24^a^	45.18 ± 10.84^a^	52.15 ± 8.79^c^	55.68 ± 7.88^c^	43.87 ± 10.73^c^	49.28 ± 10.17^c^
Physical	50.39 ± 10.67	44.77 ± 11.00^a^	38.86 ± 12.19^a^	43.23 ± 10.97^c^	45.27 ± 10.98^c^	37.87 ± 11.98^c^	41.94 ± 12.34^c^

Note: The table is based on a nonimputed dataset. Data presented as mean ± SD or frequencies (percentages).

a *P* < .001; *P*-values are based on independent *t*-test.

b *P* < .05; *P*-values are based on χ^2^ test.

c *P* < .001; *P*-values are based on one-way analysis of variance.

d *P* < .001; *P*-values are based on χ^2^ test.

At baseline, 933 participants reported not lonely. Of these, 228 (13.1% of the total) became lonely after 12 months, while 705 (40.6%) remained not lonely. Among the 802 participants who were lonely at baseline, 605 (34.9%) remained lonely, while 197 (11.4%) were no longer lonely at follow-up.

### Loneliness and frailty at baseline and follow-up

Table 2 shows the baseline and follow-up frailty scores, including overall, physical, psychological and social frailty, based on overall loneliness at baseline and changes in overall loneliness over 12 months. Compared to those who were not overall lonely at baseline, participants who were lonely had significantly higher scores for overall (6.89 ± 3.14), physical (3.76 ± 2.25), psychological (1.73 ± 1.12) and social (1.39 ± 0.86) frailty at follow-up.

**Table 2:** Loneliness and overall, physical, psychological and social frailty at baseline and follow-up (*n* = 1735)

Groups of loneliness	Baseline	Follow-up
Baseline overall loneliness	Overall frailty score	
Not lonely	3.64 ± 2.48^a^	3.48 ± 2.62^a^
Lonely	6.86 ± 2.98^a^	6.89 ± 3.14^a^
12-month change in overall loneliness		
Not lonely to lonely	4.69 ± 2.44^b^	5.90 ± 2.80^b^
Continued not lonely	3.30 ± 2.40^b^	3.16 ± 2.48^b^
Continued lonely	7.24 ± 2.94^b^	7.26 ± 3.18^b^
Lonely to not lonely	5.70 ± 2.83^b^	4.65 ± 2.77^b^
	Physical frailty score	
Baseline overall loneliness		
Not lonely	2.26 ± 1.82^a^	2.12 ± 1.94^a^
Lonely	3.78 ± 2.17^a^	3.76 ± 2.25^a^
12-month change in overall loneliness		
Not lonely to lonely	2.83 ± 1.76^b^	3.22 ± 2.10^b^
Continued not lonely	2.07 ± 1.80^b^	1.96 ± 1.86^b^
Continued lonely	4.00 ± 2.17^b^	3.97 ± 2.67^b^
Lonely to not lonely	3.10 ± 2.05^b^	2.71 ± 2.08^b^
	Psychological frailty score	
Baseline overall loneliness		
Not lonely	0.74 ± 0.91^a^	0.73 ± 0.94^a^
Lonely	1.61 ± 1.01^a^	1.73 ± 1.12^a^
12-month change in overall loneliness		
Not lonely to lonely	1.04 ± 0.97^b^	1.51 ± 1.15^b^
Continued not lonely	0.65 ± 0.87^b^	0.64 ± 0.89^b^
Continued lonely	1.72 ± 1.00^b^	1.81 ± 1.11^b^
Lonely to not lonely	1.28 ± 0.96^b^	1.06 ± 1.03^b^
	Social frailty score	
Baseline overall loneliness		
Not lonely	0.64 ± 0.70^a^	0.63 ± 0.72^a^
Lonely	1.47 ± 0.89^a^	1.39 ± 0.86^a^
12-month change in overall loneliness		
Not lonely to lonely	0.82 ± 0.77^b^	1.17 ± 0.76^b^
Continued not lonely	0.58 ± 0.67^b^	0.57 ± 0.69^b^
Continued lonely	1.52 ± 0.88^b^	1.48 ± 0.88^b^
Lonely to not lonely	1.31 ± 0.87^b^	0.88 ± 0.78^b^

Note: Data presented as mean ± SD; a higher score represents a higher level of frailty.

a *P* < .001; *P*-values are based on independent *t*-test.

b *P* < .001; *P*-values are based on one-way analysis of variance.

Supplementary Table S1 presents frailty scores stratified by baseline emotional and social loneliness. Participants who were emotionally lonely at baseline had higher follow-up frailty scores: overall (7.05 ± 3.24), physical (3.76 ± 2.27), psychological (1.80 ± 1.13) and social (1.48 ± 0.87) than those not emotionally lonely. Similarly, socially lonely participants had higher scores: overall (7.12 ± 3.30), physical (4.04 ± 2.27), psychological (1.71 ± 1.15) and social (1.37 ± 0.94) compared to those not socially lonely.

Regarding changes in overall loneliness (Table 2), participants who remained not lonely had the lowest frailty scores, while those who remained lonely had the highest scores at follow-up. Participants who transitioned from not lonely to lonely had higher frailty scores at follow-up compared to baseline. Conversely, those who shifted from lonely to not lonely had lower follow-up frailty scores than at baseline. All *P*-values ≤.05.

### Unidirectional associations between loneliness and frailty at follow-up

Table 3 displays the unidirectional associations between loneliness dimensions (overall loneliness at baseline and 12-month changes in loneliness) and frailty (overall, physical, psychological and social) at follow-up. Supplementary Table S2 provides similar associations for baseline emotional and social loneliness.

**Table 3:** Multivariate linear regression models: loneliness status and follow-up scores of overall, physical, psychological and social frailty (*n* = 1735)

Loneliness status			Baseline overall loneliness		12-month change in overall loneliness				
			Lonely vs. not lonely	Adjusted *R*^2^, %	Continued not lonely (Ref)	Not lonely to lonely	Continued lonely	Lonely to not lonely	Adjusted *R*^2^, %
12-month follow-up frailty score	Overall frailty	Crude model ^a^	**0.43 (2.60, 3.20)**	18.50%		**0.28 (2.31, 3.20)**	**0.60 (3.86, 4.50)**	**0.15 (1.09, 2.03)**	30.20%
		Adjusted model ^b^	0.04 (−0.005, 0.53)	57.40%		**0.17 (1.31, 2.00)**	**0.18 (0.97, 1.59)**	−0.009 (−0.47, 0.27)	60.70%
	Physical frailty	Crude model ^a^	**0.32 (1.24, 1.66)**	10.40%		**0.19 (0.95, 1.60)**	**0.44 (1.81, 2.28)**	**0.12 (0.49, 1.18)**	16.10%
		Adjusted model ^c^	0.02 (−0.10, 0.27)	52.00%		**0.09 (0.36, 0.86)**	**0.09 (0.21, 0.66)**	−0.004 (−0.30, 0.24)	53.00%
	Psychological frailty	Crude model ^a^	**0.36 (0.71, 0.93)**	12.50%		**0.25 (0.69, 1.02)**	**0.50 (1.09, 1.32)**	**0.12 (0.27, 0.61)**	21.60%
		Adjusted model ^d^	0.04 (−0.02, 0.20)	40.30%		**0.16 (0.39, 0.68)**	**0.16 (0.27, 0.52)**	−0.003 (−0.16, 0.14)	43.10%
	Social frailty	Crude model ^a^	**0.36 (0.55, 0.71)**	12.70%		**0.24 (0.49, 0.74)**	**0.51 (0.84, 1.02)**	**0.10 (0.16, 0.42)**	21.90%
		Adjusted model ^e^	0.04 (−0.009, 0.15)	47.80%		**0.19 (0.39, 0.59)**	**0.22 (0.32, 0.50)**	−0.03 (−0.20, 0.01)	53.00%

Note: Effect estimates are standardised linear regression coefficients and 95% confidence intervals. Numbers reported in bold represent *P* < .05.

a No covariates were adjusted.

b Adjusted for baseline age, sex, education, country, alcohol use, exercise, multimorbidity, physical and mental HR-QoL and intervention condition.

c Adjusted for baseline psychological frailty, age, sex, education, country, alcohol use, exercise, multimorbidity, physical and mental HR-QoL and intervention condition.

d Adjusted for baseline physical frailty, age, sex, education, country, alcohol use, exercise, multimorbidity, physical and mental HR-QoL and intervention condition.

e Adjusted for baseline physical frailty, age, sex, education, country, alcohol use, exercise, multimorbidity, physical and mental HR-QoL and intervention condition

Participants who were lonely at baseline had higher frailty scores across all domains at follow-up compared to those who were not lonely. However, these associations were not significant after adjusting for covariates and baseline frailty scores. Regarding social loneliness, those who were socially lonely at baseline had significantly higher follow-up frailty scores: overall (β = 0.07, 95% CI: 0.22, 0.78), physical (β = 0.07, 95% CI: 0.13, 0.53) and social (β = 0.05, 95% CI: 0.008, 0.18), after adjusting for covariates and baseline frailty scores.

Regarding changes in overall loneliness, participants who were not lonely at baseline but became lonely by follow-up (β = 0.17, 95% CI: 1.31, 2.00), and those who remained lonely (β = 0.18, 95% CI: 0.97, 1.59) had significantly higher overall scores. For physical frailty, those who transitioned from not lonely to lonely (β = 0.09, 95% CI: 0.36, 0.86) and those who remained lonely (β = 0.10, 95% CI: 0.23, 0.67 also had significantly higher physical scores at follow-up. For psychological frailty, participants who became lonely (β = 0.16, 95% CI: 0.41, 0.70) or remained lonely (β = 0.17, 95% CI: 0.28, 0.54) had higher psychological scores at follow-up. For social frailty, those who transitioned to lonely or continued being lonely had higher scores: β = 0.19 (95% CI: 0.39, 0.59) and β = 0.23 (95% CI: 0.32, 0.51), respectively. All results were significant after adjusting for covariates and baseline frailty.

### Bidirectional association between overall loneliness and overall, physical, psychological and social frailty

**Figure 1:**
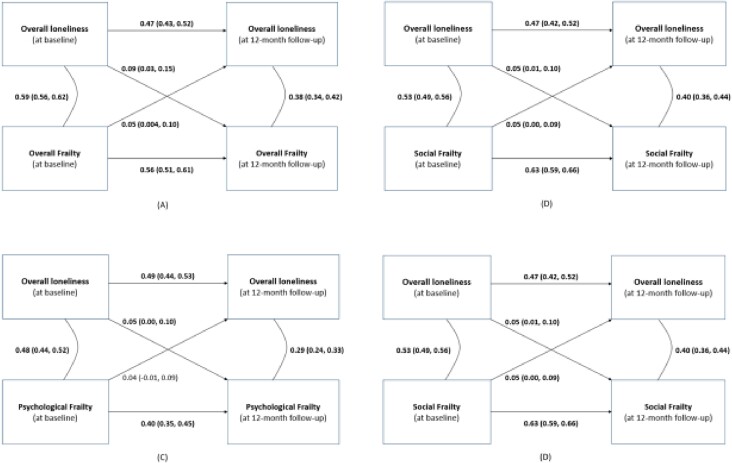
Bidirectional associations between loneliness and frailty. (A) Bidirectional association between overall loneliness and overall frailty. Values represent standardised linear regression coefficients (β, 95% confidence intervals). These coefficients represent the effect size in the model as well and are considered baseline covariates. Numbers reported in bold represent *P* < .05. *n* = 1785, model fit: Root Mean Square Error of Approximation (RMSEA) = 0.191, CFI = 0.665, TLI = 0.258. Wald test comparing lagged pathways: *P* = .0. Adjusted covariates: age, sex, country, education, alcohol use (yes/no), exercise (less/more), multimorbidity (score), HRQoL (mental and physical score) and intervention (yes/no). (B) Bidirectional association between overall loneliness and physical frailty. Values represent standardised linear regression coefficients (β, 95% confidence intervals). These coefficients represent the effect size in the model and are considered baseline covariates. Numbers reported in bold represent *P* < .05. *n* = 1785, model fit: RMSEA = 0.196, CFI = 0.578, TLI = 0.071. Wald test comparing lagged pathways: *P* = .21. Adjusted covariates: age, sex, country, education, alcohol use (yes/no), exercise (less/more), multimorbidity (score), HRQoL (mental and physical, score), intervention (yes/no) and psychological frailty (baseline score). (C) Bidirectional association between overall loneliness and psychological frailty. Values represent standardised linear regression coefficients (β, 95% confidence intervals). These coefficients represent the effect size in the model and are considered baseline covariates. Numbers reported in bold represent *P* < .05. *n* = 1785, model fit: RMSEA = 0.164, CFI = 0.642, TLI = 0.212. Wald test comparing lagged pathways: *P* < .05. Adjusted covariates: age, sex, country, education, alcohol use (yes/no), exercise (less/more), multimorbidity (score), HRQoL (mental and physical score), intervention (yes/no) and physical frailty (baseline score). (D) Bidirectional association between overall loneliness and social frailty. Values represent standardised linear regression coefficients (β, 95% confidence intervals). These coefficients represent the effect size in the model and are considered baseline covariates. *n* = 1785, model fit: RMSEA = 0.138, CFI = 0.757, TLI = 0.464. Wald test comparing lagged pathways: *P* < .05. Adjusted covariates: age, sex, country, education, alcohol use (yes/no), exercise (less/more), multimorbidity (score), HRQoL (mental and physical, score), intervention (yes/no) and physical frailty (baseline score).

The results of the cross-lagged models are presented in Figure 1.

Figure 1A demonstrates that higher levels of overall loneliness at baseline were associated with higher levels of overall frailty at follow-up (β = 0.09, 95% CI: 0.03, 0.15). Conversely, higher levels of overall frailty at baseline were associated with higher levels of overall loneliness at follow-up (β = 0.05, 95% CI: 0.004, 0.10). Both associations were significant, with the effect from loneliness to frailty being stronger than the reversed direction (Wald test: *P* < .05).

Figure 1C shows that higher levels of overall loneliness at baseline were associated with higher levels of psychological frailty at follow-up (β = 0.05, 95% CI: 0.00, 0.10); however, the reverse association was not significant.

Figure 1D shows that higher levels of overall loneliness at baseline were associated with higher levels of social frailty at follow-up (β = 0.05, 95% CI: 0.01, 0.10). Also, higher levels of social frailty at baseline were associated with higher levels of overall loneliness at follow-up (β = 0.05, 95% CI: 0.00, 0.09).

### Interaction analyses

The results of the interaction analyses are detailed in Supplementary Table S3. The interaction between country and baseline emotional loneliness concerning social frailty was statistically significant (*P* < .025).

Stratified analyses (Supplementary Table S4) revealed that the association between emotional loneliness and social frailty was significant only among participants from Spain (OR = 0.12, 95% CI: 0.02, 0.12).

## Discussion

This study explored the relationship between loneliness and frailty, examining both emotional and social dimensions of loneliness, as well as changes in overall loneliness and their associations with overall, physical, psychological and social frailty. We found that baseline social loneliness was significantly associated with higher levels of overall, physical and social frailty at follow-up. Bidirectional associations were observed between overall loneliness and both overall and social frailty but not with physical or psychological frailty. Additionally, our study highlighted that loneliness, especially social loneliness, is an independent risk factor for overall frailty and its three domains among older adults.

Consistent with previous studies [38, 46], our findings show that older age, female sex, lower education, less exercise, a higher number of chronic conditions and lower mental and physical HRQoL are associated with loneliness. These associations can be explained by various factors: older age and chronic conditions can restrict physical mobility and social interactions, increasing feelings of loneliness; sex differences in social roles and networks may contribute to the higher prevalence of loneliness among females; lower education and less exercise are often linked to fewer social opportunities and reduced social engagement, further increasing the risk of loneliness [47, 48]. However, we did not observe a significant interaction effect of sex on the relationship between loneliness and frailty, suggesting that the impact of loneliness on frailty may not differ substantially between sexes. This finding underscores the need for further research to explore this relationship in greater detail. Furthermore, while the association between emotional loneliness and social frailty was found to vary by country, significant results were only observed in Spain. This highlights the potential influence of regional factors on the relationship between emotional loneliness and social frailty, suggesting that contextual variables may shape these associations. Further studies are needed to investigate these country-specific differences and their implications for targeted interventions.

The results of our study indicate that loneliness, particularly social loneliness, may be an independent risk factor for overall, physical, psychological and social frailty among older adults. Loneliness could lead to unhealthy behaviours such as poor eating habits, reduced physical activity and fewer health-promoting practices [1, 8, 49], which contribute to adverse physical health conditions such as cardiovascular disease, diabetes and chronic obstructive pulmonary disease [50]. Additionally, inadequate social support may result in decreased access to healthcare or delayed treatment for health issues [51], potentially increasing the risk of frailty.

The finding that participants who were not lonely at baseline but became lonely at follow-up and those who were continuously lonely had higher levels of frailty at follow-up suggests that the impact of loneliness on frailty increases over time. This could be due to the psychological distress caused by loneliness, which negatively affects both mental and physical health outcomes, potentially leading to increased frailty [52]. For example, a study by Perissinotto *et al*. [53] found that an increase in loneliness over 6 years was associated with greater declines in physical function in older adults.

The study found bidirectional associations between overall loneliness and both overall and social frailty but not between overall loneliness and physical frailty. Additionally, higher levels of overall loneliness at baseline were linked to greater psychological frailty at follow-up, although the reverse was not observed. These findings can be explained by several factors. First, social frailty is closely related to social isolation and loneliness, as it involves a decline in social participation and support, which may contribute to increased overall loneliness [50]. In contrast, physical frailty is often more closely related to physical health and functional impairments than social factors [8]. This may explain the lack of a bidirectional association between overall loneliness and physical frailty. However, loneliness is a well-established risk factor for depression and anxiety, which can impact psychological frailty [38, 54]. These findings highlight the importance of preventive measures and interventions aimed at maintaining social support and engagement among older adults.

### Strengths and limitations

A strength of our study is its comprehensive approach, examining not only overall loneliness and frailty but also their emotional and social dimensions, as well as physical, psychological and social frailty. This broad perspective provides a detailed understanding of the associations between these conditions. Additionally, the study explores both unidirectional and bidirectional associations between various aspects of loneliness and frailty, making it unique in using a large population-based sample across multiple European countries.

However, there are limitations to consider. First, the reliance on self-reported questionnaires may introduce recall bias. Future research could benefit from using electronic devices for data collection. Second, handling missing data through listwise deletion led to the exclusion of 109 participants. This approach, although simple, can introduce bias if the missing data are not random, which may in turn affect the generalizability of our findings. Alternative methods, such as multiple imputation or maximum likelihood estimation, should be considered in future studies. Third, although the study identifies bidirectional associations between overall loneliness and both overall and social frailty, causality cannot be inferred from this analysis. Future research should include additional waves of data collection to explore causal relationships, with studies incorporating three or more waves of evaluation recommended [55].

## Conclusion

Our research indicates that loneliness, particularly social loneliness, independently contributes to the risk of overall, physical, psychological and social frailty in older adults. Addressing loneliness is essential for preventing and reducing frailty, with an emphasis on social loneliness. Future interventions should focus on enhancing social support, increasing engagement in physical activities and promoting healthy behaviours to mitigate loneliness and reduce frailty risks among older adults.

## Supplementary Material

aa-24-0059-File002_afae210
