# Supplementary material for: The association between loneliness and frailty among community-dwelling older adults in five European countries: a longitudinal study
Source: Age Ageing. 2024 Oct 10;53(10):afae210. doi: 10.1093/ageing/afae210 (PMC11465174; doi:10.1093/ageing/afae210)
Supplement: aa-24-0059-File002_afae210 [file aa-24-0059-file002_afae210.pdf]

Supplementary Table S1. Baseline emotional and social loneliness and overall, physical, psychological, and social frailty.

Baseline loneliness status	Baseline frailty score	Follow-up frailty score
Baseline emotional loneliness	Overall frailty	
Not lonely	4.10 ± 2.68 [a]	4.32 ± 3.05 [a]
Lonely	7.61 ±2.84 [a]	7.05 ± 3.24 [a]
Baseline social loneliness		
Not lonely	4.42 ±2.84 [a]	4.38 ± 3.04 [a]
Lonely	7.06 ± 3.17 [a]	7.12 ±3.30 [a]
Baseline emotional loneliness	Physical frailty	
Not lonely	2.50±1.94 [a]	2.56 ±2.14 [a]
Lonely	4.09±2.14 [a]	3.76 ±2.27 [a]
Baseline social loneliness		
Not lonely	2.60 ± 1.97 [a]	2.50 ± 2.09
Lonely	3.94 ± 2.24 [a]	4.04 ± 2.27
Baseline emotional loneliness	Psychological frailty	
Not lonely	0.86 ±0.94	0.97 ± 1.06 [a]
Lonely	1.83 ± 0.97	1.80 ± 1.13 [a]
Baseline social loneliness		
Not lonely	0.96 ± 0.98 [a]	1.02 ± 1.09 [a]

Lonely	1.66 ± 1.05 [a]	1.71 ± 1.15 [a]
Baseline emotional loneliness	Social frailty	
Not lonely	0.75 ± 0.76 [a]	0.80 ±0.80 [a]
Lonely	1.69 ±0.84 [a]	1.48 ± 0.87 [a]
Baseline social loneliness		
Not lonely	0.86 ± 0.82 [a]	0.86 ± 0.81 [a]
Lonely	1.46 ± 0.93 [a]	1.37 ± 0.94 [a]

Note: Data presented as mean ±SD; a higher score represents a higher level of frailty.

[a] P < 0.001; P values are based on independent t test.

[b] P < 0.001; P values are based on one-way analysis of variance.

Supplementary Table S2. Multivariate Linear Regression Models: emotional and social loneliness and follow-up scores of overall, physical, psychological, social frailty.

Baseline loneliness status	12-month follow-up frailty score							
	Overall frailty		Physical frailty		Psychological frailty		Social frailty	
	Crude model [a]	Adjusted model [b]	Crude model [a]	Adjusted model [c]	Crude model [a]	Adjusted model [d]	Crude model [a]	Adjusted model [e]
Emotional loneliness								
Lonely V.S. not lonely	0.38 (2.48, 3.16)	-0.006 (-0.34, 0.25)	0.26 (1.02, 1.50)	-0.04 (-0.40, 0.006)	0.35 (0.77, 1.00)	0.04 (-0.01, 0.22)	0.35 (0.59, 0.77)	0.01 (-0.06, 0.12)
Adjusted R^2, %	14.5%	57.3%	6.5%	52.1%	12.0%	40.3%	12.1%	47.7%
Social loneliness								
Lonely V.S. not lonely	0.37 (2.46, 3.16)	0.07 (0.22, 0.78)	0.31 (1.34, 1.82)	0.07 (0.13, 0.53)	0.28 (0.60, 0.85)	0.03 (-0.04, 0.20)	0.26 (0.42, 0.61)	0.05 (0.008, 0.18)
Adjusted R^2, %	13.8%	57.6%	9.8%	52.3%	7.6%	40.3%	6.6%	47.8%

Note: Effect estimates are standardized linear regression coefficients and 95% confidence intervals.

[a] No covariates were adjusted.

[b] Adjusted for baseline age, sex, education, country, alcohol use, exercise, multi-morbidity, physical and mental HR-QoL, and intervention condition.

[c] Adjusted for baseline psychological frailty, age, sex, education, country, alcohol use, exercise, multi-morbidity, physical and mental HR-QoL, and intervention condition.

[d] Adjusted for baseline physical frailty, age, sex, education, country, alcohol use, exercise, multi-morbidity, physical and mental HR-QoL, and intervention condition.

[e] Adjusted for baseline physical frailty, age, sex, education, country, alcohol use, exercise, multi-morbidity, physical and mental HR-QoL, and intervention condition.

Supplementary Table S3. P-values of Interaction Terms in Multivariable Linear Regression Models for Follow-up Overall, Physical, Psychological, and Social Frailty.

Interaction Items	Overall Frailty	Physical Frailty	Psychological Frailty	Social Frailty
Sex * loneliness at baseline	0.11	0.12	0.66	0.27
Sex * emotional loneliness at baseline	0.15	0.23	0.18	0.98
Sex * social loneliness at baseline	0.89	0.38	0.30	0.86
Country * loneliness at baseline	0.13	0.48	0.18	0.11
Country * emotional loneliness at baseline	0.07	0.56	0.18	**0.01**
Country * social loneliness at baseline	0.40	0.88	0.11	0.71

P-values were derived by separately adding the interaction terms to the multivariable linear regression models for socio-demographic characteristics and overall frailty and three domains among 1735 participants of the UHCE study. Bonferroni correction for multivariable linear regression was applied (P=0.05/2 = 0.025).

Supplementary Table S4. Stratified Associations by Country: Baseline Emotional Loneliness and Follow-up Overall, Physical, Psychological, Social Frailty.

Baseline Emotional loneliness	12-Month Follow-up Social Frailty Score	
	Crude model [a]	Adjusted model [b]
The Netherlands	**0.50 (0.33, 0.53)**	0.06 (-0.07, 0.18)
Greece	**0.30 (0.12, 0.36)**	-0.11 (-0.23, 0.05)
Croatia	**0.32 (0.18, 0.34)**	0.04 (-0.06, 0.12)
Spain	**0.43 (0.28, 0.44)**	**0.12 (0.02, 0.18)**
The United Kingdom	**0.47 (0.39, 0.57)**	0.03 (-0.06, 0.12)

Note: Effect estimates are standardized linear regression coefficients and 95% confidence intervals. Numbers reported in bold represent $P<0.05$.

a No covariates were adjusted.

b Adjusted for baseline physical frailty, age, sex, education, alcohol use, exercise, multi-morbidity, physical and mental HR-QoL, and intervention condition
